# The effect of epidermal growth factor receptor mutation on adjuvant chemotherapy with tegafur/uracil for patients with completely resected, non-lymph node metastatic non-small cell lung cancer (> 2 cm): a multicenter, retrospective, observational study as exploratory analysis of the CSPOR-LC03 study

**DOI:** 10.1093/jjco/hyae073

**Published:** 2024-09-11

**Authors:** Tomohiro Miyoshi, Keiju Aokage, Shun-ichi Watanabe, Hiroyuki Ito, Noriaki Sakakura, Mingyon Mun, Motohiro Yamashita, Yasuhisa Ohde, Tadashi Aoki, Wataru Nishio, Masataka Taguri, Masahiro Tsuboi

**Affiliations:** Division of Thoracic Surgery, Department of Thoracic Oncology, National Cancer Center Hospital East, Chiba, Japan; Division of Thoracic Surgery, Department of Thoracic Oncology, National Cancer Center Hospital East, Chiba, Japan; Division of Thoracic Surgery, National Cancer Center Hospital, Tokyo, Japan; Department of Thoracic Surgery, Kanagawa Cancer Center, Kanagawa, Japan; Department of Thoracic Surgery, Aichi Cancer Center Hospital, Nagoya, Japan; Department of Thoracic Surgical Oncology, Cancer Institute Hospital, Japanese Foundation for Cancer Research, Tokyo, Japan; Department of Thoracic Surgery, Shikoku Cancer Center, Ehime, Japan; Division of Thoracic Surgery, Shizuoka Cancer Center Hospital, Shizuoka, Japan; Department of Chest Surgery, Niigata Cancer Center Hospital, Niigata, Japan; Department of Chest Surgery, Hyogo Cancer Center, Hyogo, Japan; Department of Health Data Science, Tokyo Medical University, Tokyo, Japan; Division of Thoracic Surgery, Department of Thoracic Oncology, National Cancer Center Hospital East, Chiba, Japan

**Keywords:** lung cancer, adjuvant chemotherapy, tegafur/uracil, epidermal growth factor receptor

## Abstract

**Background:**

The use of adjuvant osimertinib for epidermal growth factor receptor (EGFR) mutants is expected to expand to earlier stage I in the future, potentially competing with the current standard of care, oral tegafur/uracil (UFT), in Japan. However, the effect of EGFR mutation status on the therapeutic effect of UFT remains unclear. This study was conducted as an exploratory analysis of a retrospective observational study that investigated the real-world data of postoperative adjuvant chemotherapy in Japan (CSPOR-LC03).

**Methods:**

Between 2008 and 2013, 1812 patients with completely resected adenocarcinoma diagnosed as pathologic stage I (T1 > 2 cm, TNM classification, sixth edition) who have maintained organ function, and no history of other cancers were included. The primary endpoint was the 5-year disease-free survival (DFS) rate, and we compared this rate between four groups classified based on the administration of adjuvant UFT and EGFR mutation status.

**Results:**

Of the 933 (51%) patients with EGFR mutations, 394 underwent adjuvant UFT therapy. Of the 879 (49%) patients without EGFR mutations, 393 underwent adjuvant UFT therapy. The 5-year DFS of UFT+/EGFR+ and UFT−/EGFR+ patients were 82.0 and 87.1%, respectively, and those of UFT+/EGFR− and UFT−/EGFR− patients were 80.0 and 86.9%, respectively. DFS was significantly worse in the UFT+ group than in the UFT− group (*P* = 0.015). Adjuvant UFT therapy was not an independent prognostic factor for DFS, regardless of the EGFR mutation status.

**Conclusion:**

In pathologic stage I (>2 cm) lung adenocarcinomas with EGFR mutation, the survival benefit of adjuvant UFT was not observed.

## Teaser

In pathologic stage I (>2 cm) lung adenocarcinomas with EGFR mutation, the survival benefit of adjuvant chemotherapy with UFT was not observed.

## Introduction

Lung cancer is the leading cause of cancer-related deaths worldwide. According to the annual report of the Japanese Association for Thoracic Surgery, over 42 107 lung resections were performed for patients with primary lung cancer in Japan in 2016 [1] The initial standard treatments for lung cancer of clinical stages I and II are lobectomy and lymphadenectomy, respectively. Adjuvant chemotherapy is performed based on the postoperative pathological stage, aiming to eliminate micrometastatic lesions to minimize postoperative recurrence risk. The standard treatment for pathological stage I (> 2 cm) and IIA non-small cell lung cancer (NSCLC) in Japan is adjuvant chemotherapy using an oral tegafur/uracil combination agent (UFT). UFT is a fluoropyrimidine drug that inhibits dihydro-pyrimidine dehydrogenase (DPD), a rate-limiting enzyme in the catabolism of fluorouracil and is associated with drug resistance. It has demonstrated a 5% improvement in a 5-year survival rate [2–6].

The efficacy of epidermal growth factor receptor (EGFR)-tyrosine kinase inhibitors (EGFR-TKIs) in treating EGFR mutant advanced lung cancer has been established [7] Furthermore, attempts are being made to explore the use of EGFR-TKIs as adjuvant therapy for earlier stages of lung cancer. The ADAURA trial was conducted as a randomized phase III study comparing osimertinib, a third-generation EGFR-TKI, with a placebo as adjuvant therapy in patients with stages IB–IIIA EGFR mutant NSCLC. This study demonstrated the significant efficacy of osimertinib regarding disease-free survival (DFS) and overall survival (OS) [8–10] The subgroup analysis of the ADAURA trial also suggested the usefulness of adjuvant chemotherapy with osimertinib for pathological stage IB (> 3 cm) (4-year DFS: 80% versus 59%; hazard ratio [HR]: 0.41; 95% confidence interval [CI]: 0.23–0.69). However, in the ADAURA trial, which used a placebo control, Japanese patients with pathological stage IB (> 3 cm), for whom oral UFT was the standard adjuvant treatment, were not enrolled. Thus, in Japan, data on the efficacy and safety of osimertinib in the seventh edition of stage IB patients are lacking. Therefore, which is the standard of care for this stage of the disease, osimertinib or UFT, remains undefined.

Furthermore, the usefulness of adjuvant therapy using osimertinib for earlier pathological stage IA lung cancer with EGFR mutation is being verified in the ADAURA2 trial [11] The positioning of UFT in relation to osimertinib may become an issue with regard to the target stage of the ADAURA2 trial, too.

Although a small-scale study has reported that adjuvant chemotherapy with UFT in patients with EGFR mutation had no effect on survival prolongation [12,13], the therapeutic efficacy of UFT in lung cancer with EGFR mutations has not been fully elucidated in large-scale studies.

CSPOR-LC03 was a large-scale, retrospective, multicenter observational study conducted to understand real-world data on adjuvant chemotherapy for NSCLC in Japan. The study demonstrated a survival benefit of UFT in a high-risk subgroup (ground-glass opacity [GGO] absent and a total tumor size >3 cm). Therefore, we planned an exploratory analysis of CSPOR-LC03 to elucidate the effect of EGFR mutations on adjuvant chemotherapy with UFT.

## Patients and methods

This is a physician-initiated clinical research project supported by an agreement between AstraZeneca K.K., the Public Health Research Center, and the principal investigator. This study was conducted under a subcontract agreement between the collaborative research institute and the Public Health Research Center. The institutional review board of each participating hospital approved the study protocol before initiating the study (Institutional Review Board Number: National Cancer Center Hospital 2021-215, 15 February 2022). This study was registered with the University Hospital Medical Information Network-CTR (UMIN000047443).

Because the study does not involve invasive or interventional procedures, and all the samples and information used are residual samples and information obtained from medical practice in the past, based on the ‘Ethical Guidelines for Medical and Health Research Involving Human Subjects,’ the requirement for informed consent was waived by disclosing the implementation of this study on an easily accessible website, etc., in order to give the patients an opportunity to refuse to participate. The matters to be disclosed include information on the significance, objectives, and methodology of the study, names of participating institutions, and contact information for inquiries, complaints, etc.

### Background of the CSPOR-LC03 study

Between November 2008 and December 2013, the Japan Clinical Oncology Group conducted a randomized phase III trial (JCOG 0707) comparing standard UFT and experimental S-1 (TS-1®: a combination agent of tegafur, gimeracil, and oteracil potassium) as adjuvant chemotherapy for patients with NSCLC (> 2 cm) without lymph node metastases [14] This trial did not demonstrate a superior effect of S-1 over UFT regarding survival prolongation. As the patients enrolled in JCOG0707 were highly selected, CSPOR-LC03, a large-scale, retrospective, multicenter observational study, was conducted to identify the real-world situation on adjuvant chemotherapy [15,16] Researchers participating in JCOG0707 retrospectively reviewed medical records for patients who were not enrolled in JCOG0707 during its enrollment period [17].

Eligibility criteria included the following: (1) pathologically diagnosed NSCLC, excluding low-grade malignant tumors such as carcinoid, mucoepidermoid carcinoma, and adenoid cystic carcinoma; (2) pathological stage I disease with a tumor size >2 cm (T1 > 2 cm and T2 in sixth TNM edition); (3) pathologically confirmed complete (R0) resection; (4) lobectomy or larger lung resection has been performed; (5) standard hilar or mediastinal lymph node dissection has been performed; (6) no prior treatment before lung resection; and (7) not previously enrolled in JCOG0707. The supplementary material details the eligibility criteria for CSPOR-LC03 and JCOG0707.

CSPOR-LC03 revealed that approximately 75% of patients who met the eligibility criteria for JCOG0707, namely with maintained organ function and a history of no other cancers, were not enrolled for reasons such as patients’ enrollment decline or physicians’ direction that adjuvant therapy is unnecessary.

### Analysis of population and methodology

Patients with completely resected pathological Stage I (T1 > 2 cm and T2 in sixth TNM edition) NSCLC eligible for the JCOG0707 but excluded from the trial during its enrollment period were eligible for this exploratory analysis.

**Figure 1:**
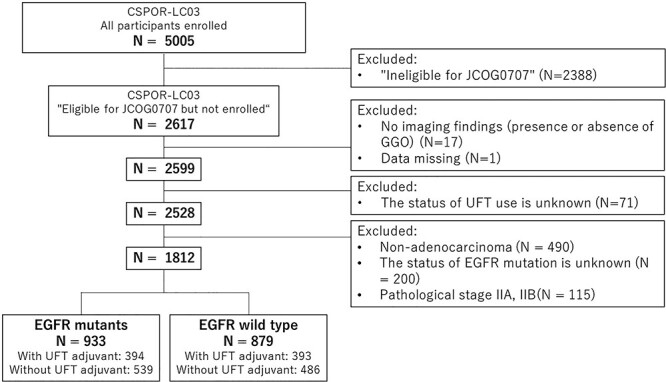
CONSORT flow diagram. CSPOR, comprehensive support project for oncology research; JCOG, Japan clinical oncology group; GGO, ground-grass opacity; UFT, oral tegafur/uracil combination agent; EGFR, epidermal growth factor receptor.

In CSPOR-LC03, data from 5005 patients with completely resected NSCLC were collected from 34 institutions in Japan. Through a retrospective analysis of patient demographic characteristics, tumor profiles, and postoperative treatments, 2599 patients were classified as ‘eligible for JCOG0707 but were not actually enrolled.’ Of the 2599 patients, 71 were those for whom whether they received adjuvant UFT therapy was unknown, 1 patient with insufficient data, 490 with non-adenocarcinoma, and 115 with pathological stages IIA and IIB were excluded. Thus, the remaining 1812 patients became the participants of this observational study (Fig. 1). Additional gene analysis using the Cobas® EGFR Mutation Test v2 was performed using surgical specimens for patients whose EGFR mutation statuses were unknown. More detailed data on clinicopathological findings, such as lymphovascular invasion, for all participants were collected.

The primary endpoint is the 5-year DFS rate, and the secondary endpoint is the 5-year OS rate. DFS is defined as the period (days) from the operation to disease progression or death from any cause, whichever occurred first. It is censored on the last day on which survival is confirmed without any evidence of these events. OS is defined as the period (days) from the operation to death from any cause and is censored at the last day on which survival is confirmed.

This study aimed to clarify whether the EGFR mutation status is a predictor of treatment response to adjuvant UFT therapy and is a prognostic factor. DFS and OS were compared between four groups classified based on the presence or absence of adjuvant UFT chemotherapy and EGFR mutations. We identified prognostic factors for recurrence and death through univariable and multivariable analyses. Subsequently, we analyzed the interaction between EGFR mutation and UFT therapy.

### Statistical analysis

Fisher’s exact and Wilcoxon rank sum tests were performed to evaluate differences in the patient characteristics for categorical and continuous variables, respectively. To clarify whether EGFR mutation status is a predictor of treatment response to adjuvant UFT therapy and is a prognostic factor, Kaplan–Meier survival curves of the DFS and OS were generated for the four groups classified based on the presence or absence of adjuvant UFT chemotherapy and EGFR mutations. We estimated the 5-year DFS/OS and their 95% CIs for each group. In addition, these four groups were compared using a log-rank test as a univariable analysis. We performed univariable and multivariable analyses using a Cox proportional hazards model to identify prognostic factors for recurrence and death using clinicopathological factors, including the presence or absence of adjuvant UFT chemotherapy and EGFR mutations. Subsequently, we analyzed the interaction between EGFR mutation and adjuvant UFT. In addition, we examined the impact of UFT on DFS and OS by EGFR mutation status using inverse probability of treatment weighting to mitigate potential differences in baseline characteristics between groups. The following factors were used for adjustment: age, sex, lymph node dissection, total tumor size, GGO component, pathologic stage, pleural invasion, vascular invasion, and lymphatic permeation.

The numbers of patients in the four groups (UFT+/EGFR+, UFT+/EGFR−, UFT−/EGFR+, and UFT−/EGFR−) were 394, 393, 539, and 486, respectively. When the prognostic difference between the UFT+/EGFR+ and UFT+/EGFR− groups in the 5-year DFS rate was set at 5% (UFT+/EGFR+ group: 87%; UFT+/EGFR− group: 92%), the statistical power was calculated using the Wald test of a multivariable Cox proportional hazards model. The calculation resulted in a statistical power of 83.4% and 74.3% at a one- and two-sided significance level of 5%, respectively.

**Table 1:** Patients’ characteristics based on EGFR mutation and the administration of UFT.

Characteristics	EGFR mutant *n* = 933			EGFR wild type *n* = 879		
	With UFT *n* = 394 (%)	Without UFT *n* = 539 (%)	*P* value	With UFT *n* = 393 (%)	Without UFT *n* = 486 (%)	*P* value
Age						
< 70	273 (69)	322 (60)	0.003	275 (70)	272 (56)	< 0.0001
≥ 70	121 (31)	217 (40)		118 (30)	214 (44)	
Sex						
Male	147 (37)	176 (33)	0.14	235 (60)	273 (56)	0.28
Female	247 (63)	363 (67)		158 (40)	213 (44)	
Surgical procedure						
Lobectomy	394 (100)	536 (99)	0.14	393 (100)	483 (99)	0.12
Other	0 (0)	3 (1)		0 (0)	3 (1)	
Lymph node dissection						
ND2a-1	203 (52)	350 (65)	< 0.0001	186 (47)	283 (58)	0.001
ND2a-2	191 (49)	189 (35)		207 (53)	203 (42)	
Total tumor size (cm)						
≦ 3 cm	190 (48)	383 (71)	< 0.0001	163 (42)	313 (64)	< 0.0001
> 3 cm	204 (52)	156 (29)		230 (59)	173 (36)	
GGO						
Present	228 (58)	378 (70)	0.0001	155 (39)	261 (54)	0.0001
Absent	166 (42)	161 (30)		238 (61)	225 (46)	
Pathological stage						
IA	167 (42)	388 (72)	< 0.0001	127 (32)	299 (62)	< 0.0001
IB	227 (58)	151 (28)		266 (68)	187 (39)	
Pleural invasion						
Present	96 (25)	67 (13)	< 0.0001	114 (31)	72 (16)	< 0.0001
Absent	298 (75)	472 (87)		279 (69)	414 (84)	
Vascular invasion						
Present	114 (30)	91 (18)	< 0.0001	142 (38)	127 (27)	0.001
Absent	280 (70)	448 (82)		251 (62)	359 (63)	
Lymphatic permeation						
Present	84 (22)	78 (15)	0.008	65 (17)	84 (18)	0.79
Absent	310 (78)	461 (85)		328 (83)	402 (82)	

EGFR, epidermal growth factor receptor; UFT, oral tegafur/uracil combination agent; ND, node dissection; GGO, ground-grass opacity

## Results

The median duration of follow-up of participants was 5.8 years (interquartile range [IQR]: 5.0–7.1 years). Of the 1812 patients, 831 (46%) were men, and the median preoperative age was 67 years (IQR: 61–72 years). As tumors >5 cm in total size were not included in this study, the study participants corresponded to stage I (> 2 cm) in the seventh edition of TNM. Nine hundred and thirty-three (51%) patients had EGFR mutations, and 394 patients of them underwent UFT adjuvant therapy. Of the 879 (49%) patients without EGFR mutation, 393 patients underwent UFT adjuvant therapy. The main reasons why UFT was not administered to the 1025 patients were as follows: (1) 623 (61%) patients preferred observation; (2). physicians determined that adjuvant therapy was not necessary for 316 (31%) patients. Among patients with EGFR mutations, 94 (24%) received UFT treatment for less than 6 months, 49 (12%) for 6 months to less than 1 year, and 251 (64%) for 1 year or more. In patients without EGFR mutations, the corresponding figures were 89 (23%), 47 (12%), and 257 (65%), respectively. The patients’ characteristics were compared based on the EGFR mutation status and UFT adjuvant therapy (Table 1). In EGFR mutant and non-mutant groups, older age, ND2a-2 (versus ND2a-1), larger total tumor size of >3 cm (versus ≤3 cm), pure solid nodule (versus nodule with GGO), pathological stage IB (versus IA), pleural indentation-positive (versus negative), and vascular invasion-positive (versus negative) were significantly frequent in the UFT adjuvant group compared with the observation group. Lymphatic permeation was significantly frequent in the UFT adjuvant group in only patients with EGFR mutations.

Supplemental Table 1 presents patient background based on EGFR mutation status. Compared with EGFR non-mutants, in EGFR mutants, women (versus men), ND-2a-1 (versus ND2a-2), smaller total tumor size of ≤3 cm (versus >3 cm), nodule with GGO (versus pure solid nodule), pathological stage IA (versus IB), pleural indentation-negative (versus positive), and vascular invasion-negative (versus positive) were significantly frequent. Supplemental Table 2 lists the EGFR mutation subtypes.

**Figure 2:**
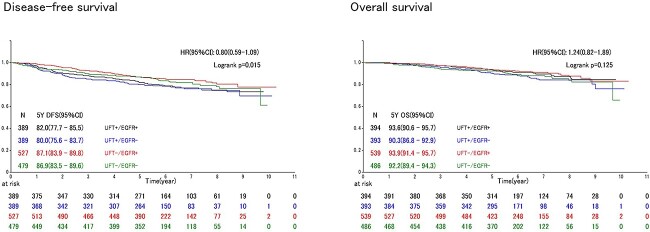
DFS and OS among all patients. UFT, oral tegafur/uracil combination agent; EGFR, epidermal growth factor receptor; GGO, ground-grass opacity.

Figure 2 illustrates the DFS and OS of the four groups based on the EGFR mutation status and UFT adjuvant therapy. UFT+ patients had poorer DFS than UFT− patients regardless of the EGFR mutation status. The 5-year DFS of UFT+/EGFR+ and UFT−/EGFR+ patients were 82.0 (95% CI, 77.7–85.5%) and 87.1% (95% CI, 83.9–89.8%), respectively, and those of UFT+/EGFR− and UFT−/EGFR− patients were 80.0 (95% CI, 75.6–83.7%) and 86.9% (95% CI, 83.5%–89.6%), respectively. DFS was worse in the UFT+ group than in the UFT− group, regardless of the EGFR mutation status (*P* = 0.015). In OS analysis, no significant difference was observed between the four groups (*P* = 0.125).

**Table 2A:** Univariable and multivariable analyses for the risk factors for DFS.

		Univariable		Multivariable	
Variable	Reference	HR (95% CI)	*P* value	HR (95% CI)	*P* value
EGFR mutation, positive	Negative	0.889 (0.716–1.105)	0.29	1.171 (0.926–1.481)	0.19
With UFT	Without UFT	1.404 (1.130–1.744)	0.002	0.987 (0.778–1.252)	0.91
Age, ≥ 70	< 70	1.183 (0.952–1.469)	0.13	1.021 (0.810–1.288)	0.86
Sex, male	Female	1.400 (1.125–1.743)	0.003	1.333 (1.060–1.677)	0.014
Lymph node dissection, ND2a-2	ND2a-1	1.216 (0.978–1.511)	0.078	1.100 (0.877–1.380)	0.41
Total tumor size, cm	1 cm increase	1.460 (1.262–1.689)	< 0.0001	1.145 (0.960–1.365)	0.13
Pathological stage, IB	IA	2.379 (1.899–2.981)	< 0.0001	1.287 (0.917–1.805)	0.14
GGO, present	Absent	0.284 (0.224–0.360)	< 0.0001	0.436 (0.334–0.568)	< 0.0001
Pleural invasion, present	Absent	3.295 (2.625–4.135)	< 0.0001	1.538 (1.151–2.053)	0.004
Vascular invasion, present	Absent	4.002 (3.199–5.007)	< 0.0001	2.173 (1.665–2.836)	< 0.0001
Lymphatic permeation, Ppresent	Absent	2.592 (2.039–3.295)	< 0.0001	1.371 (1.057–1.779)	0.017

EGFR, epidermal growth factor receptor; UFT, oral tegafur/uracil combination agent; ND, node dissection; GGO, ground-grass opacity; HR, hazard ratio; CI, confidence interval

We performed univariable and multivariable analyses to detect the prognostic factors in DFS (Table 2A) and OS (Table 2B). In DFS, with adjuvant UFT (HR, 1.404; 95% CI, 1.130–1.744: *P* = 0.002), male sex, total tumor size, pleural invasion positive, lymphatic permeation positive, vascular invasion positive, nodule with GGO, and pathological stage IB were significant predictive factors in the univariable analysis. In the multivariable analysis, male sex (HR, 1.333; 95% CI, 1.060–1.677: *P* = 0.014), pleural invasion positive (HR, 1.538; 95% CI, 1.151–2.053: *P* = 0.004), lymphatic permeation positive (HR, 1.371; 95% CI, 1.057–1.779: *P* = 0.017), vascular invasion positive (HR, 2.173; 95% CI, 1.665–2.836: *P* < 0.0001), and nodule with GGO (HR, 0.436; 95% CI, 0.334–0.568: *P* < 0.0001) were significant. However, the significance of adjuvant UFT disappeared (HR, 0.987; 95% CI, 0.778–1.252: *P* = 0.91).

**Table 2B:** Univariable and multivariable analyses for the risk factors for overall survival.

		Univariable		Multivariable	
Variable	Reference	HR (95% CI)	*P* value	HR (95% CI)	*P* value
EGFR mutation, positive	Negative	0.716 (0.536–0.956)	0.024	0.923 (0.675–1.262)	0.62
With UFT	Without UFT	1.129 (0.846–1.506)	0.41	0.808 (0.588–1.110)	0.19
Age, ≥ 70	< 70	1.616 (1.207–2.163)	0.001	1.433 (1.051–1.956)	0.023
Sex, male	Female	1.692 (1.268–2.257)	0.0004	1.613 (1.195–2.177)	0.002
Lymph node dissection, ND2a-2	ND2a-1	0.958 (0.716–1.282)	0.77	0.896 (0.662–1.213)	0.48
Total tumor size, cm	1 cm increase	1.494 (1.242–1.797)	< 0.0001	1.308 (1.031–1.659)	0.027
Pathological stage, IB	IA	1.969 (1.466–2.644)	< 0.0001	1.079 (0.687–1.696)	0.74
GGO, present	Absent	0.339 (0.249–0.463)	< 0.0001	0.570 (0.403–0.807)	0.002
Pleural invasion, present	Absent	2.394 (1.761–3.255)	< 0.0001	1.216 0.827–1.787)	0.32
Vascular invasion, present	Absent	3.922 (2.914–5.279)	< 0.0001	2.517 (1.763–3.593)	< 0.0001
Lymphatic permeation, present	Absent	2.590 (1.897–3.537)	< 0.0001	1.399 (0.993–1.972)	0.055

EGFR, epidermal growth factor receptor; UFT, oral tegafur/uracil combination agent; ND, node dissection; GGO, ground-grass opacity; HR, hazard ratio; CI, confidence interval

**Figure 3:**
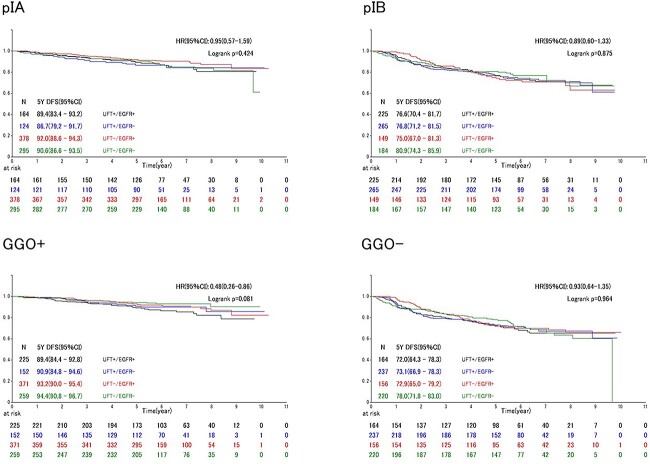
DFS among subgroup patients. UFT, oral tegafur/uracil combination agent; EGFR, epidermal growth factor receptor; GGO, ground-grass opacity.

In OS, EGFR mutation-positive (HR, 0.716; 95% CI, 0.536–0.956: *P* = 0.024), age ≥ 70, male sex, total tumor size, pleural invasion positive, lymphatic permeation positive, vascular invasion positive, nodule with GGO, and pathological stage IB were significant predictive factors in the univariable analysis. In the multivariable analysis, age ≥ 70 (HR, 1.433; 95% CI, 1.051–1.956: *P* = 0.023), male sex (HR, 1.613; 95% CI, 1.195–2.177: *P* = 0.002), total tumor size (HR, 1.308; 95% CI, 1.031–1.659: *P* = 0.027), vascular invasion positive (HR, 2.517; 95% CI, 1.763–3.593: *P* < 0.0001), and nodule with GGO (HR, 0.570; 95% CI, 0.403–0.807: *P* < 0.0001) were significant. However, the significance of EGFR mutation-positive disappeared (HR, 0.923; 95% CI, 0.675–1.262: *P* = 0.62). UFT adjuvant therapy and EGFR mutation status were not independent prognostic factors for either DFS or OS.

**Figure 4:**
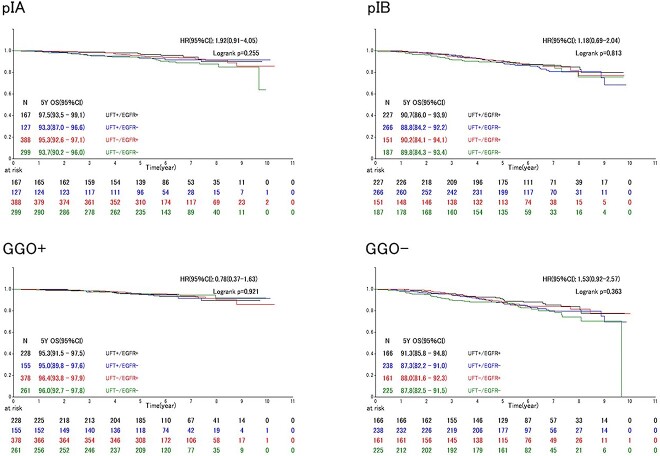
Overall survival among subgroup patients. UFT, oral tegafur/uracil combination agent; EGFR, epidermal growth factor receptor; GGO, ground-grass opacity.

No significant difference was observed between the four groups in any subgroup analyses based on pathological stage IA/IB or with/without GGO in DFS and OS (Figs 3 and 4). Univariable and multivariable analyses restricted to the higher-risk subgroups, without GGO and tumor diameter > 3 cm, also showed no effect of UFT, regardless of EGFR mutation (Supplemental Tables 3 and 4).

Supplemental Figure 1 illustrates the impact of UFT on DFS and OS by EGFR mutation status using inverse probability of treatment weighting. Despite adjusting potential differences in baseline characteristics between groups (Supplemental Table 5), the efficacy of adjuvant UFT was not demonstrated for either DFS or OS, regardless of EGFR mutation status.

## Discussion

An *in vitro* study revealed that EGFR mutant cells were less sensitive to fluorouracil than were EGFR wild-type cells [18] High expression of DPD, a 5-fluorouracil degrading enzyme, correlated with EGFR mutation in adenocarcinoma cells and tissues. This correlation weakened the sensitivity of 5-fluorouracil [19] Pemetrexed, another anti-metabolite drug, has also been suggested to have limited efficacy in patients with EGFR mutations [20] Some retrospective studies have reported limited benefits of adjuvant therapy with UFT in patients with EGFR mutation-positive NSCLC [13,18] To our knowledge, this study is the first to examine the effect of UFT based on the EGFR mutation status in a large-scale population. Another strength of this study is that it only included patients in good general condition who would meet the eligibility criteria for a prospective clinical trial. This study aimed to clarify whether the EGFR mutation status is a predictor of treatment response to UFT. However, adjuvant therapy with UFT did not affect DFS and OS, regardless of the EGFR mutation status.

In JCOG 0707, which had the same eligibility criteria as the present study, 70.7% of patients received UFT treatment for at least one year, which is comparable to the results of the present study (64.5%). In addition, dose reduction and treatment completion rates in JCOG0707 were reported to be 20.1 and 60.0%, respectively. Although this study did not collect this information, we consider that treatment adherence in the present study is not significantly different from previous reports.

DFS was employed as the primary endpoint in this study because OS is substantially affected by treatment following relapse. Univariable analysis of DFS revealed a worse prognosis in the UFT+ group than in the UFT− group, regardless of the EGFR mutation status. When observing a positive survival effect of adjuvant therapy in retrospective studies, the influence of patient selection bias should be considered, as adjuvant therapy is more likely to be administered to fit patients. However, the opposite trend was observed in this study. Because this study exclusively enrolled physically low-risk patients eligible for JCOG0707—namely, those deemed suitable candidates for adjuvant UFT—this outcome was ascribed to another selection bias that UFT was preferentially administered to patients with an elevated risk of recurrence. Consequently, the significance of postoperative UFT disappeared after adjusting for patient backgrounds including lymphovascular invasion in the multivariable analysis.

Tsutani et al. reported no significant difference in recurrence-free survival between patients with and without adjuvant UFT in pathologically high-risk stage I EGFR-positive lung adenocarcinoma, particularly T1c/T2a or those with lymphovascular invasion [13] However, they found a benefit of UFT in the EGFR mutation-negative group in contrast to the results of this study. In this study, although the number of cases in the subgroup analysis was limited and may be underpowered to detect differences, the consistent lack of evidence regarding the benefit of UFT in multivariable analyses focusing on the high-risk group suggests that the benefit of adjuvant UFT on DFS may be limited, regardless of the EGFR mutation status. Another approach, survival analysis adjusted for patient background with inverse probability of treatment weighting also failed to demonstrate the efficacy of adjuvant UFT. To date, no randomized controlled trials have investigated the significance of adjuvant UFT over placebo with DFS as the primary endpoint, but the failure to reproduce the results of previous prospective clinical trials and meta-analyses may be attributed to improvements in diagnostic accuracy and treatment effectiveness, and increased number of GGO lesions with good prognosis.

Regarding the lack of effect of UFT on OS regardless of the EGFR mutation status, the result of the original research of CSPOR-LC03 must be mentioned. CSPOR-LC03 failed to demonstrate a significant OS prolongation with adjuvant UFT in the JCOG 0707-eligible population, however, significance was observed only in a high-risk subgroup characterized by the absence of GGO and a total tumor size >3 cm. This study could not reveal an OS benefit of UFT even in the high-risk subgroup. One of the reasons for this might be a lack of power due to a further reduction in the number of cases because this study further excluded non-adenocarcinomas from the CSPOR-LC03 population. However, multivariable analyses consistently did not show UFT efficacy, even when focusing on the high-risk group, suggesting that the benefit of adjuvant UFT on OS may be limited, regardless of the EGFR mutation status.

The prognostic role of EGFR mutation remains unclear, given the inconsistent results from previous studies [21–24] Aokage et al. reported that in cases of early-stage lung cancer without GGO, DFS exhibited no significant difference based on the EGFR mutation status. However, the cumulative incidence of recurrence demonstrated a tendency to be elevated in patients with EGFR mutation compared to those without. Moreover, OS was better in patients with EGFR mutation than those without [21] In this study, the univariable analysis of OS also demonstrated a more favorable prognosis in patients with EGFR mutation, irrespective of adjuvant UFT. As EGFR-TKI is an effective key drug for patients with EGFR mutations after recurrence, retrospective studies tend to indicate a better OS in patients with EGFR mutations. However, in this study, the multivariable analysis adjusting for patient backgrounds eliminated the significance of this difference, suggesting that the improved prognosis in patients with EGFR mutations might be due to its favorable patient characteristics rather than the effect of EGFR-TKI.

In the stage IB subgroup in the ADAURA trial, 5-year DFS and OS in the osimertinib group were 78 (95% CI, 67–86%) and 94% (95% CI, 86–97%), respectively. In contrast, in this study, 5-year DFS and OS in the stage IB subgroup of the UFT+/EGFR+ population were 76.6 (95% CI, 70.4–81.7%) and 90.7% (95% CI, 86.0–93.9%), respectively. These results are not simply comparable; hence, prospective studies are needed to verify whether osimertinib or UFT is the more appropriate agent for postoperative adjuvant therapy for EGFR mutation-positive NSCLC in this pathological stage in Japan.

This study has some limitations. First, although the sample size is adequate, the number of cases in the subgroup analysis was limited and potentially underpowered to detect certain effects. Second, as a retrospective study, it was susceptible to various biases, including physician judgment regarding UFT use. Third, although DFS is set as the primary endpoint in this study, the schedule for the postoperative follow-up was not prescribed and standardized. Fourth, there is no detailed information on postrecurrence treatment. The administration rate of EGFR-TKI after recurrence can have a substantial effect on OS.

In conclusion, in pathologic stage I (> 2 cm) lung adenocarcinomas with EGFR mutation, no survival benefit of adjuvant chemotherapy with UFT was observed. Further investigation through prospective observational studies is warranted.

### Meeting presentation

This paper was presented during the 40th annual meeting of the Japanese Association for Chest Surgery, July 13–14, 2023, Niigata, Japan (10902).

## Supplementary Material

Supplemental_Figure1_hyae073

Supplemental_Table1_hyae073

Supplemental_Table2_hyae073

Supplemental_Table3_hyae073

Supplemental_Table4_hyae073

Supplemental_Table5_hyae073

Supplemental_material_hyae073

## Data sharing

Qualified researchers can request access to study documents (including the clinical study report, the study protocol with any amendments, and a statistical analysis plan) that support the methods and findings reported in this manuscript.

## Notes

**Clinical registration number**: UMIN000047443
