# Supplementary material for: The effect of epidermal growth factor receptor mutation on adjuvant chemotherapy with tegafur/uracil for patients with completely resected, non-lymph node metastatic non-small cell lung cancer (> 2 cm): a multicenter, retrospective, observational study as exploratory analysis of the CSPOR-LC03 study
Source: Jpn J Clin Oncol. 2024 Sep 11;54(11):1185–93. doi: 10.1093/jjco/hyae073 (PMC11532619; doi:10.1093/jjco/hyae073)
Supplement: Supplemental_Figure1_hyae073 [file supplemental_figure1_hyae073.docx]

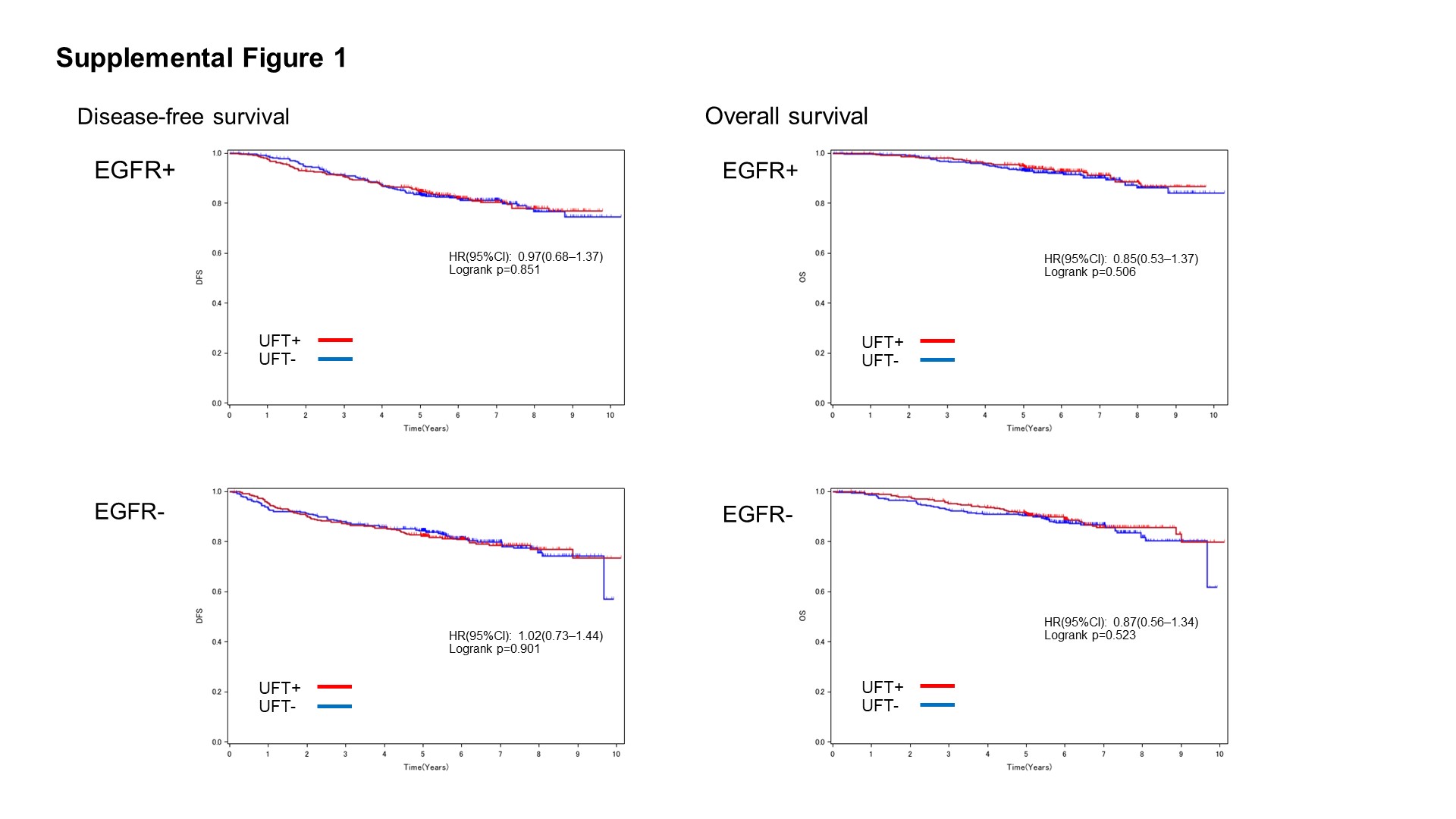


Supplemental Figure 1: The impact of UFT on disease-free survival and overall survival by EGFR mutation status using inverse probability of treatment weighting to mitigate potential differences in baseline characteristics of patients. UFT, oral tegafur/uracil combination agent; EGFR, epidermal growth factor receptor
