# Supplementary material for: The effect of epidermal growth factor receptor mutation on adjuvant chemotherapy with tegafur/uracil for patients with completely resected, non-lymph node metastatic non-small cell lung cancer (> 2 cm): a multicenter, retrospective, observational study as exploratory analysis of the CSPOR-LC03 study
Source: Jpn J Clin Oncol. 2024 Sep 11;54(11):1185–93. doi: 10.1093/jjco/hyae073 (PMC11532619; doi:10.1093/jjco/hyae073)
Supplement: Supplemental_Table1_hyae073 [file supplemental_table1_hyae073.docx]

**Supplemental Table 1: Patients’ characteristics based on the EGFR mutation status**

|  | EGFR mutant  n = 933 (%) | EGFR wild type  n = 879 (%) | *P* value |
| --- | --- | --- | --- |
| Age |  |  |  |
| < 70 | 595 (64) | 547 (62) | 0.50 |
| ≥ 70 | 338 (36) | 332 (38) |  |
| Sex |  |  |  |
| Male | 323 (35) | 508 (58) | < 0.0001 |
| Female | 610 (65) | 371 (42) |  |
| Surgical procedure |  |  |  |
| Lobectomy | 930 (99) | 876 (99) | 0.94 |
| Other | 3 (1) | 3 (1) |  |
| Lymph node dissection |  |  |  |
| ND2a-1 | 553 (59) | 469 (53) | 0.011 |
| ND2a-2 | 380 (41) | 410 (47) |  |
| Total tumor size (cm) |  |  |  |
| ≦ 3 cm | 573 (61) | 476 (54) | 0.0018 |
| > 3 cm | 360 (39) | 403 (46) |  |
| GGO |  |  |  |
| Present | 606 (65) | 463 (53) | < 0.0001 |
| Absent | 327 (35) | 416 (47) |  |
| Pathological stage |  |  |  |
| IA | 555 (59) | 426 (48) | < 0.0001 |
| IB | 378 (41) | 453 (52) |  |
| Pleural invasion |  |  |  |
| Present | 163 (17) | 186 (21) | 0.047 |
| Absent | 770 (83) | 693 (79) |  |
| Vessel invasion |  |  |  |
| Present | 205 (22) | 269 (31) | < 0.0001 |
| Absent | 728 (78) | 610 (69) |  |
| Lymphatic permeation |  |  |  |
| Present | 162 (17) | 149 (17) | 0.82 |
| Absent | 771 (83) | 730 (83) |  |

EGFR, epidermal growth factor receptor; UFT, oral tegafur/uracil combination agent; ND, node dissection; GGO, ground-grass opacity
