# Supplementary material for: The effect of epidermal growth factor receptor mutation on adjuvant chemotherapy with tegafur/uracil for patients with completely resected, non-lymph node metastatic non-small cell lung cancer (> 2 cm): a multicenter, retrospective, observational study as exploratory analysis of the CSPOR-LC03 study
Source: Jpn J Clin Oncol. 2024 Sep 11;54(11):1185–93. doi: 10.1093/jjco/hyae073 (PMC11532619; doi:10.1093/jjco/hyae073)
Supplement: Supplemental_Table2_hyae073 [file supplemental_table2_hyae073.docx]

**Supplemental Table 2: EGFR mutation subtypes**

| Subtypes | EGFR mutant  n = 933 | | |
| --- | --- | --- | --- |
|  | With UFT  n = 394 (%) | Without UFT  n = 539 (%) | *P* value |
| Exon19 deletion | 167 (42) | 215 (40) | 0.44 |
| L858R | 181 (46) | 272 (50) | 0.17 |
| G719X | 15 (4) | 13 (2) | 0.22 |
| L861Q | 11 (3) | 10 (2) | 0.34 |
| Exon 20 insertion | 4 (1) | 9 (2) | 0.40 |
| S768I | 2 (1) | 0 (0) | 0.10 |
| Other | 14 (4) | 20 (4) | 0.90 |

EGFR, epidermal growth factor receptor; UFT, oral tegafur/uracil combination agent
