# Supplementary material for: The effect of epidermal growth factor receptor mutation on adjuvant chemotherapy with tegafur/uracil for patients with completely resected, non-lymph node metastatic non-small cell lung cancer (> 2 cm): a multicenter, retrospective, observational study as exploratory analysis of the CSPOR-LC03 study
Source: Jpn J Clin Oncol. 2024 Sep 11;54(11):1185–93. doi: 10.1093/jjco/hyae073 (PMC11532619; doi:10.1093/jjco/hyae073)
Supplement: Supplemental_Table3_hyae073 [file supplemental_table3_hyae073.docx]

**Supplemental Table 3A: Univariable and multivariable analyses for risk factors for disease-free survival in subgroup analysis (EGFR mutation-positive, GGO absent, total tumor size > 3 cm, n = 108)**

|  |  | Univariable | | Multivariable | |  |
| --- | --- | --- | --- | --- | --- | --- |
| Variable | Reference | HR (95% CI) | P value | HR (95% CI) | P value | |
| With UFT | Without UFT | 1.004 (0.526–1.915) | 0.99 | 1.174 (0.531–2.594) | 0.69 | |
| Age, ≥ 70 | < 70 | 0.709 (0.366–1.373) | 0.31 | 0.691 (0.337–1.415) | 0.31 | |
| Sex, Male | Female | 1.800 (0.939–3.451) | 0.0766 | 2.209 (1.030–4.739) | 0.042 | |
| Lymph node dissection, ND2a-2 | ND2a-1 | 1.166 (0.614–2.212) | 0.64 | 1.231 (0.604–2.507) | 0.57 | |
| Total tumor size, cm | 1 cm increase | 1.348 (0.741–2.450) | 0.33 | 1.368 (0.661–2.831) | 0.40 | |
| Pleural invasion, Present | Absent | 1.573 (0.818–3.025) | 0.14 | 1.275 (0.637–2.552) | 0.49 | |
| Vessel invasion, Present | Absent | 2.114 (1.084–4.124) | 0.028 | 2.156 (1.012–4.596) | 0.047 | |
| Lymphatic permeation, Present | Absent | 1.096 (0.532–2.258) | 0.80 | 0.878 (0.375–2.053) | 0.76 | |

EGFR, epidermal growth factor receptor; UFT, oral tegafur/uracil combination agent; ND, node dissection; GGO, ground-grass opacity; HR, hazard ratio; CI, confidence interval

**Supplemental Table 3B: Univariable and multivariable analyses for risk factors for disease-free survival in subgroup analysis (EGFR mutation-negative, GGO absent, total tumor size > 3 cm, n = 202)**

|  |  | Univariable | | Multivariable | |  |
| --- | --- | --- | --- | --- | --- | --- |
| Variable | Reference | HR (95% CI) | P value | HR (95% CI) | P value | |
| With UFT | Without UFT | 1.025 (0.609–1.725) | 0.93 | 0.984 (0.567–1.709) | 0.96 | |
| Age, ≥ 70 | < 70 | 1.045 (0.639–1.711) | 0.86 | 0.943 (0.558–1.596) | 0.83 | |
| Sex, Male | Female | 0.619 (0.363–1.054) | 0.078 | 0.578 (0.329–1.015) | 0.056 | |
| Lymph node dissection, ND2a-2 | ND2a-1 | 0.810 (0.499–1.313) | 0.39 | 1.130 (0.663–1.924) | 0.65 | |
| Total tumor size, cm | 1 cm increase | 1.288 (0.862–1.923) | 0.22 | 1.212 (0.775–1.895) | 0.40 | |
| Pleural invasion, Present | Absent | 2.113 (1.287–3.470) | 0.003 | 1.591 (0.940–2.694) | 0.084 | |
| Vessel invasion, Present | Absent | 3.639 (2.054–6.448) | < 0.0001 | 2.891 (1.536–5.441) | 0.001 | |
| Lymphatic permeation, Present | Absent | 2.096 (1.256–3.497) | 0.005 | 1.335 (0.747–2.387) | 0.33 | |

EGFR, epidermal growth factor receptor; UFT, oral tegafur/uracil combination agent; ND, node dissection; GGO, ground-grass opacity; HR, hazard ratio; CI, confidence interval
