# Supplementary material for: The effect of epidermal growth factor receptor mutation on adjuvant chemotherapy with tegafur/uracil for patients with completely resected, non-lymph node metastatic non-small cell lung cancer (> 2 cm): a multicenter, retrospective, observational study as exploratory analysis of the CSPOR-LC03 study
Source: Jpn J Clin Oncol. 2024 Sep 11;54(11):1185–93. doi: 10.1093/jjco/hyae073 (PMC11532619; doi:10.1093/jjco/hyae073)
Supplement: Supplemental_Table4_hyae073 [file supplemental_table4_hyae073.docx]

**Supplemental Table 4A: Univariable and multivariable analyses for the risk factors for overall survival in subgroup analysis (EGFR mutation-positive, GGO absent, total tumor size > 3 cm, n = 108)**

|  |  | Univariable | | Multivariable | |  |
| --- | --- | --- | --- | --- | --- | --- |
| Variable | Reference | HR (95% CI) | P value | HR (95% CI) | P value | |
| With UFT | Without UFT | 0.844 (0.332–2.143) | 0.72 | 0.913 (0.270–3.091) | 0.88 | |
| Age, ≥ 70 | < 70 | 0.954 (0.369–2.466) | 0.92 | 1.022 (0.349–2.988) | 0.97 | |
| Sex, Male | Female | 2.368 (0.897–6.256) | 0.082 | 2.660 (0.773–9.147) | 0.12 | |
| Lymph node dissection, ND2a-2 | ND2a-1 | 0.614 (0.242–1.558) | 0.30 | 0.600 (0.190–1.893) | 0.38 | |
| Total tumor size, cm | 1 cm increase | 1.621 (0.798–3.291) | 0.18 | 1.281 (0.387–4.242) | 0.68 | |
| Pleural invasion, Present | Absent | 2.012 (0.745–5.435) | 0.17 | 1.251 (0.447–3.500) | 0.67 | |
| Vessel invasion, Present | Absent | 9.067 (2.119–38.791) | 0.003 | 9.293 (2.004–43.105) | 0.004 | |
| Lymphatic permeation, Present | Absent | 1.555 (0.576–4.199) | 0.38 | 1.115 (0.295–4.213) | 0.87 | |

EGFR, epidermal growth factor receptor; UFT, oral tegafur/uracil combination agent; ND, node dissection; GGO, ground–grass opacity; HR, hazard ratio; CI, confidence interval

**Supplemental Table 4B: Univariable and multivariable analyses for risk factors for overall survival in subgroup analysis (EGFR mutation-negative, GGO absent, total tumor size > 3 cm, n = 202)**

|  |  | Univariable | | Multivariable | |  |
| --- | --- | --- | --- | --- | --- | --- |
| Variable | Reference | HR (95% CI) | P value | HR (95% CI) | P value | |
| With UFT | Without UFT | 0.886 (0.461–1.702) | 0.72 | 0.807 (0.416–1.566) | 0.53 | |
| Age, ≥ 70 | < 70 | 1.063 (0.567–1.995) | 0.85 | 1.030 (0.541–1.958) | 0.93 | |
| Sex, Male | Female | 0.881 (0.462– 1.681) | 0.70 | 0.885 (0.454–1.724) | 0.72 | |
| Lymph node dissection, ND2a-2 | ND2a-1 | 0.855 (0.465–1.571) | 0.61 | 1.082 (0.560–2.089) | 0.81 | |
| Total tumor size, cm | 1 cm increase | 1.102 (0.663–1.833) | 0.71 | 1.074 (0.604–1.910) | 0.81 | |
| Pleural invasion, Present | Absent | 2.044 (1.102–3.791) | 0.023 | 1.506 (0.784–2.894) | 0.22 | |
| Vessel invasion, Present | Absent | 3.198 (1.595–6.412) | 0.001 | 2.585 (1.200–5.573) | 0.015 | |
| Lymphatic permeation, Present | Absent | 2.037 (1.075–3.860) | 0.029 | 1.337 (0.660–2.710) | 0.42 | |

EGFR, epidermal growth factor receptor; UFT, oral tegafur/uracil combination agent; ND, node dissection; GGO, ground-grass opacity; HR, hazard ratio; CI, confidence interval
