# Supplementary material for: The effect of epidermal growth factor receptor mutation on adjuvant chemotherapy with tegafur/uracil for patients with completely resected, non-lymph node metastatic non-small cell lung cancer (> 2 cm): a multicenter, retrospective, observational study as exploratory analysis of the CSPOR-LC03 study
Source: Jpn J Clin Oncol. 2024 Sep 11;54(11):1185–93. doi: 10.1093/jjco/hyae073 (PMC11532619; doi:10.1093/jjco/hyae073)
Supplement: Supplemental_Table5_hyae073 [file supplemental_table5_hyae073.docx]

**Supplemental Table5. Adjusted patients’ background after inverse probability of treatment weighting**

| Characteristics | Total  n = 1738 | | | |
| --- | --- | --- | --- | --- |
|  | EGFR mutant  n = 900 | | EGFR wild type  n = 838 | |
|  | With UFT  n = 383 | Without UFT  n = 517 | With UFT  n = 374 | Without UFT  n = 464 |
| Age |  |  |  |  |
| < 70 | 62.7% | 63.4% | 61.6% | 61.9% |
| ≥ 70 | 37.3% | 36.6% | 38.4% | 38.1% |
| Sex |  |  |  |  |
| Male | 35.4% | 35.2% | 58.0% | 57.8% |
| Female | 64.6% | 64.8% | 42.0% | 42.2% |
| Lymph node dissection |  |  |  |  |
| ND2a-1 | 40.4% | 40.7% | 45.9% | 46.2% |
| ND2a-2 | 59.6% | 59.3% | 54.1% | 53.8% |
| Total tumor size (cm) |  |  |  |  |
| median (range) | 2.60 (1.2−5.0) | 2.70 (1.5−5.0) | 2.80 (1.5−5.0) | 2.80 (1.6−5.5) |
| mean (SD) | 2.84 (0.99) | 2.85 (0.89) | 3.03 (1.12) | 3.03 (1.02) |
| GGO |  |  |  |  |
| Present | 63.7% | 64.1% | 48.1% | 48.0% |
| Absent | 36.3% | 35.9% | 51.9% | 52.0% |
| Pathological stage |  |  |  |  |
| IA | 60.3% | 60.0% | 48.7% | 48.6% |
| IB | 39.7% | 40.0% | 51.3% | 51.4% |
| Pleural invasion |  |  |  |  |
| Present | 17.6% | 17.8% | 23.0% | 23.8% |
| Absent | 82.4% | 82.2% | 77.0% | 76.2% |
| Vascular invasion |  |  |  |  |
| Present | 18.9% | 19.0% | 17.5% | 17.6% |
| Absent | 81.1% | 81.0% | 82.5% | 82.4% |
| Lymphatic permeation |  |  |  |  |
| Present | 23.9% | 23.3% | 32.0% | 32.3% |
| Absent | 76.1% | 76.7% | 68.0% | 67.7% |

EGFR, epidermal growth factor receptor; UFT, oral tegafur/uracil combination agent; ND, node dissection; SD, standard deviation; GGO, ground-grass opacity
