# Supplementary material for: The effect of epidermal growth factor receptor mutation on adjuvant chemotherapy with tegafur/uracil for patients with completely resected, non-lymph node metastatic non-small cell lung cancer (> 2 cm): a multicenter, retrospective, observational study as exploratory analysis of the CSPOR-LC03 study
Source: Jpn J Clin Oncol. 2024 Sep 11;54(11):1185–93. doi: 10.1093/jjco/hyae073 (PMC11532619; doi:10.1093/jjco/hyae073)
Supplement: Supplemental_material_hyae073 [file supplemental_material_hyae073.docx]

**Supplementary material:** The eligibility criteria of CSPOR-LC03 and JCOG0707

[Eligibility criteria of the CSPOR-LC03 study]

(1) Histopathologically diagnosed with non-small cell lung cancer, a solid malignancy. However, low-grade tumors, such as carcinoid, mucoepidermoid carcinoma, and adenoid cystic carcinoma, are excluded.

(2) Diagnosed as pathological stage I (T1>2 cm, TNM Classification sixth edition).

(3) Pathologically confirmed complete resection.

Complete resection represents the complete macroscopic and histologic removal of lung cancer. Histologically identified residual cancer or a metastasis-positive surgical margin in lymphadenectomy constitutes an “incomplete resection.”

(4) Patients who underwent lobectomy or more extensive surgical resection during the period from the start month of each institution.

(5) Patients who underwent ND2a or a more extensive lymphadenectomy or selective lymphadenectomy.

(6) No previous treatment other than surgery.

(7) Not enrolled in clinical studies and trials related to postoperative treatments.

The patients who met the eligibility criteria for the JCOG0707 study and were not enrolled in the study are eligible for this secondary analysis. The inclusion and exclusion criteria of the JCOG0707 study are listed below.

[Eligibility criteria of JCOG0707]

1) Patients histopathologically diagnosed with non-small cell lung cancer, a solid malignancy. However, patients with low-grade tumors, such as carcinoid, mucoepidermoid carcinoma, and adenoid cystic carcinoma, are excluded.

2) Patients diagnosed with pathological stage I. (IA is defined as a maximum tumor diameter of T1>2 cm.)

3) Pathologically confirmed complete resection.

4) Patients who underwent lobectomy or more extensive surgical resection.

5) Patients who underwent ND2a or more extensive lymphadenectomy or selective lymphadenectomy.

6) No previous treatment other than surgery.

7) Performance status (ECOG) of 0 or 1.

8) Patients with adequate organ function (bone marrow, liver, kidney, and lung).

9) Aged between 20 and 80 years.

10) Patients who underwent surgery within 56 days.

11) Written informed consent to participate in this study has been obtained from the patient.

[Exclusion criteria]

1) Patients with active multiple primary cancers.

2) Patients with serious postoperative complications.

3) Patients with serious complications (interstitial pneumonia, pulmonary fibrosis, intestinal paralysis, intestinal obstruction, diarrhea, poorly controlled diabetes mellitus, liver cirrhosis, liver failure, renal failure, poorly controlled hypertension, history of myocardial infarction within 6 months, or unstable angina).

4) Patients with a history of serious drug allergy (≥Grade 3).

5) Patients requiring continuous use of flucytosine, phenytoin, or warfarin potassium.

6) Women who are pregnant, breastfeeding, possibly pregnant, or intending to become pregnant, or men who intend to father a child.

7) Patients with psychosis or psychiatric symptoms that make participation in the study difficult.

8) Patients who tested positive for human immunodeficiency virus. (However, confirming whether they test negative for serological testing is not essential).
